# Vitamin D levels and susceptibility to asthma, elevated immunoglobulin E levels, and atopic dermatitis: A Mendelian randomization study

**DOI:** 10.1371/journal.pmed.1002294

**Published:** 2017-05-09

**Authors:** Despoina Manousaki, Lavinia Paternoster, Marie Standl, Miriam F. Moffatt, Martin Farrall, Emmanuelle Bouzigon, David P. Strachan, Florence Demenais, Mark Lathrop, William O. C. M. Cookson, J. Brent Richards

**Affiliations:** 1 Centre for Clinical Epidemiology, Department of Epidemiology, Lady Davis Institute for Medical Research, Jewish General Hospital, McGill University, Montréal, Canada; 2 MRC Integrative Epidemiology Unit, School of Social & Community Medicine, University of Bristol, Bristol, United Kingdom; 3 Institute of Epidemiology I, Helmholtz Zentrum München–German Research Center for Environmental Health, Neuherberg, Germany; 4 National Heart and Lung Institute, Imperial College London, London, United Kingdom; 5 Wellcome Trust Centre for Human Genetics, University of Oxford, Oxford, United Kingdom; 6 Division of Cardiovascular Medicine, Radcliffe Department of Medicine, University of Oxford, Oxford, United Kingdom; 7 Genetic Variation and Human Diseases Unit, UMR-946, INSERM, Université Paris Diderot, Université Sorbonne Paris Cité, Paris, France; 8 Population Health Research Institute, St George's University of London, London, United Kingdom; 9 McGill University and Genome Québec Innovation Centre, Montréal, Canada; 10 Royal Brompton and Harefield NHS Foundation Trust, London, United Kingdom; 11 Departments of Medicine and Human Genetics, McGill University, Montréal, Canada; 12 Department of Twin Research and Genetic Epidemiology, King's College London, London, United Kingdom; Imperial College London, UNITED KINGDOM

## Abstract

**Background:**

Low circulating vitamin D levels have been associated with risk of asthma, atopic dermatitis, and elevated total immunoglobulin E (IgE). These epidemiological associations, if true, would have public health importance, since vitamin D insufficiency is common and correctable.

**Methods and findings:**

We aimed to test whether genetically lowered vitamin D levels were associated with risk of asthma, atopic dermatitis, or elevated serum IgE levels, using Mendelian randomization (MR) methodology to control bias owing to confounding and reverse causation. The study employed data from the UK Biobank resource and from the SUNLIGHT, GABRIEL and EAGLE eczema consortia. Using four single-nucleotide polymorphisms (SNPs) strongly associated with 25-hydroxyvitamin D (25OHD) levels in 33,996 individuals, we conducted MR studies to estimate the effect of lowered 25OHD on the risk of asthma (*n* = 146,761), childhood onset asthma (*n* = 15,008), atopic dermatitis (*n* = 40,835), and elevated IgE level (*n* = 12,853) and tested MR assumptions in sensitivity analyses. None of the four 25OHD-lowering alleles were associated with asthma, atopic dermatitis, or elevated IgE levels (*p* ≥ 0.2). The MR odds ratio per standard deviation decrease in log-transformed 25OHD was 1.03 (95% confidence interval [CI] 0.90–1.19, *p* = 0.63) for asthma, 0.95 (95% CI 0.69–1.31, *p* = 0.76) for childhood-onset asthma, and 1.12 (95% CI 0.92–1.37, *p* = 0.27) for atopic dermatitis, and the effect size on log-transformed IgE levels was −0.40 (95% CI −1.65 to 0.85, *p* = 0.54). These results persisted in sensitivity analyses assessing population stratification and pleiotropy and vitamin D synthesis and metabolism pathways. The main limitations of this study are that the findings do not exclude an association between the studied outcomes and 1,25-dihydoxyvitamin D, the active form of vitamin D, the study was underpowered to detect effects smaller than an OR of 1.33 for childhood asthma, and the analyses were restricted to white populations of European ancestry. This research has been conducted using the UK Biobank Resource and data from the SUNLIGHT, GABRIEL and EAGLE Eczema consortia.

**Conclusions:**

In this study, we found no evidence that genetically determined reduction in 25OHD levels conferred an increased risk of asthma, atopic dermatitis, or elevated total serum IgE, suggesting that efforts to increase vitamin D are unlikely to reduce risks of atopic disease.

## Abstract 2

In a Mendelian randomization analysis, J. Brent Richards and colleagues investigate possible relations between genetic elements associated with vitamin D levels and atopic susceptibility.

## Author summary

**Why was this study done?:**

- Observational epidemiological studies have reported associations of low vitamin D levels with risk of asthma, atopic dermatitis, and elevated immunoglobulin E (IgE) levels. However, these studies are susceptible to confounding and reverse causation, and thus it remains unclear whether these associations are genuine.
- The randomized controlled trials published to date on this topic have been inconclusive.
- If vitamin D insufficiency did cause atopic diseases, this would be of clinical relevance, since vitamin D insufficiency is common and safely correctable.

**What did the researchers do and find?:**

- We applied a Mendelian randomization study design, which greatly limits bias due to confounding and prevents bias due to reverse causation, to understand if vitamin D levels are associated with a higher risk of adult and pediatric asthma, atopic dermatitis, or elevated IgE levels.
- Despite high statistical power, our study showed no evidence of an unconfounded association between vitamin D and the studied outcomes.

**What do these findings mean?:**

- Our findings suggest that the previous epidemiological associations between vitamin D and atopic diseases could be due to confounding.
- Efforts to increase vitamin D levels will probably not result in decreased risk of adult and pediatric asthma, atopic dermatitis, or elevated IgE levels.

## Introduction

Atopy refers to the shared predisposition to develop allergic diseases, such as asthma and atopic dermatitis, and is characterized by increased serum immunoglobulin E (IgE) levels. Observational studies have identified a controversial association between low vitamin D status (as measured by serum 25-hydroxyvitamin D [25OHD] levels) and risk of asthma, atopic dermatitis, and elevated serum IgE levels [1–9]. If low vitamin D were a causal risk factor for atopic diseases, this would be important for public health because vitamin D insufficiency is common, affecting 42% of Americans [10], and correctable using vitamin D supplementation.

The immune system appears to play an important role in atopy pathogenesis, and vitamin D, a proposed modulator of the immune system response, may influence the development of atopic susceptibility [11]. Although two randomized controlled trials (RCTs) [12,13] and a recent Cochrane meta-analysis of RCTs for asthma [14] showed a role for vitamin D supplementation in the reduction of atopic exacerbations, other recent data do not support the benefits of vitamin D supplementation for asthma [12,13,15,16], atopic dermatitis, or IgE levels [17–19]. Given this lingering controversy, clinical practice guidelines do not support vitamin D supplementation to prevent atopic disease [1,4]. However, RCT evidence is typically of low quality in vitamin D trials because of their small sample size and limited duration; since vitamin D therapy cannot be patented, large-scale trials would rely upon the limited means of the public purse.

Given the inconclusive results from the existing RCTs and observational studies, the principles of Mendelian randomization (MR) can be applied to test the role of biomarkers in disease etiology [20]. MR uses genetic data to ascertain whether a given biomarker, such as 25OHD, is implicated in disease etiology, relying on a simple tenet: if a biomarker is etiologically involved in a disease process, then the genetic factors that influence the biomarker will influence disease risk. This established technique greatly limits confounding, since genotypes are expected to be randomly assorted at conception; further, it is free of reverse causation since genotypes are always assigned prior to the onset of disease. Thus, MR studies overcome some of the limitations of observational studies and are conceptually similar to RCTs, but provide a lifelong assessment of exposure to a biomarker, such as low 25OHD levels. Further, recent advances in genotyping enable the application of MR methods in sample sizes that are not realistic for RCTs of vitamin D therapy.

In the present study, we adopted an MR design to estimate the effect of genetically lowered 25OHD levels on atopic susceptibility, combining retrospective data from several large-scale studies of people of European descent. Our MR instruments were single-nucleotide polymorphisms (SNPs) identified by the Study of Underlying Genetic Determinants of Vitamin D and Highly Related Traits (SUNLIGHT) Consortium, the largest genome-wide association study (GWAS) published to date for 25OHD levels (*n* = 33,995) [21]. We then applied MR to test whether genetically lowered 25OHD levels influenced asthma and atopic dermatitis susceptibility and total serum IgE levels, using data from the largest GWAS meta-analyses to date—the asthma GABRIEL consortium [22] combined with the UK Biobank [23]—for a total sample size of 25,471 cases and 121,290 controls for asthma; childhood asthma in 7,047 cases and 7,961 controls from the GABRIEL consortium; 10,788 cases and 30,047 controls for atopic dermatitis in the Early Genetics and Lifecourse Epidemiology (EAGLE) Eczema Consortium [24]; and the GABRIEL consortium for IgE levels (5,888 asthma cases and 6,965 controls).

## Methods

### Ethical approval

All human studies were approved by their institutional ethics review committees, and all participants provided written consent.

### SNP selection and data sources

This study did not have a prospective analysis plan, since it was based on already available data from large GWAS meta-analyses. Specifically, we selected the lead genome-wide significant SNPs (*p*-values < 5 x 10^−8^) associated with 25OHD from the SUNLIGHT Consortium [21] as instruments in the present MR analysis.

The estimates of the effect of each SNP on 25OHD levels were obtained using data from the Canadian Multicentre Osteoporosis Study (CaMos) [25], since the effect of each SNP on 25OHD levels was not reported in the SUNLIGHT Consortium (because of the different 25OHD measurement methods used among individual cohorts) and because CaMos was among the largest replication cohorts in the SUNLIGHT Consortium, thereby providing more accurate effects of SNPs on 25OHD. The effects of these SNPs in CaMos have been previously reported [26].

To obtain precise estimates of the genome-wide significant SNPs for vitamin D levels on asthma, we tested the effect of each of these SNPs in a meta-analysis of the UK Biobank Asthma GWAS [27] with the GABRIEL consortium (S1 Text) [22].

The association between vitamin D levels and asthma may be stronger in children [28] than in adults [29]. Therefore, we conducted a separate analysis in a subsample of the GABRIEL consortium, including 15,008 individuals (7,047 cases / 7,961 controls) with childhood-onset asthma.

For atopic dermatitis, we obtained the effects of the selected SNPs from the EAGLE Eczema Consortium [24]. For naturally log-transformed total serum IgE levels, the same estimates were obtained from the GABRIEL consortium. A description of the participating studies and definitions of the asthma, atopic dermatitis, and IgE phenotypes appear in S1 Text.

### SNP validation

To validate the four SUNLIGHT 25OHD SNPs as instruments for our MR analysis, we tested them for the three MR assumptions: strong association with the exposure (25OHD); absence of association with known confounders of the exposure—outcome association; and absence of pleiotropy, where the genetic variant influences the atopic outcome through mechanisms that are independent of the vitamin D. Bias due to linkage disequilibrium (LD) and population stratification has been previously tested for the four 25OHD-associated SNPs [26].

Because of randomization of alleles at conception, confounding is greatly minimized in MR studies; however, we have examined if 25OHD-associated SNPs may influence important known confounders that may link vitamin D to common disease [30]. Specifically, these SNPs were not associated with sun exposure, time outside, physical activity, smoking, or body mass index (BMI) [30].

Pleiotropy may bias results if the chosen SNPs exert effects on asthma, atopic dermatitis, and IgE levels independently of the 25OHD levels. In this study, pleiotropy is less likely since all 25OHD-associated SNPs map to genes strongly implicated in 25OHD physiology. Nonetheless, we conducted a PubMed literature search to identify possible pleiotropic mechanisms (S1 Text).

### Statistical analysis

#### Association of SUNLIGHT SNPs with asthma, atopic dermatitis, and IgE level

We first assessed whether each SNP was associated with risk of asthma, atopic dermatitis, or elevated IgE level, applying a Bonferroni correction, where statistical significance was declared at *p* ≤ 0.05 / 4 since four SNPs were used as instruments.

#### MR estimates

We assessed the effects of the SNPs upon the four outcomes, weighting the effect of each SNP by the magnitude of its effect upon 25OHD levels. In the absence of available data on 25OHD levels in the GWAS assessing the four outcomes, the instrumental variable estimates of genetically determined odds ratios and betas were calculated by using the two-sample MR approach [31]. To provide a summary measure for the effect including all SNPs genome-wide significant for 25OHD, we combined weighted estimates using fixed effects models and used the *I*^*2*^ estimate as a measure of heterogeneity [32,33]. The effect size for the meta-analysis is reported in our results as the effect of a standard deviation (SD) change in natural log-transformed 25OHD levels, since this metric is more interpretable than an arbitrary difference. Finally, we undertook power calculations [34] to test whether our study was adequately powered to detect a clinically relevant change in the outcomes.

#### Sensitivity analyses

Our MR estimates were recalculated after exclusion of SNPs potentially influenced by pleiotropy or population stratification. We also performed a stratified MR analysis in which SNPs involved in either 25OHD synthesis or metabolism were analyzed separately [30].

## Results

### SNP selection and validation

#### SNP selection

The SUNLIGHT Consortium identified four genome-wide significant vitamin-D associated SNPs [21]: rs2282679 in *GC* (vitamin D binding protein), rs12785878 near *DHCR7* (7-dehydrocholesterol reductase), rs10741657 near *CYP2R1* (cytochrome P450 family 2 subfamily R member 1), and rs6013897 in *CYP24A1* (cytochrome P450 family 24 subfamily A member 1) (Table 1). All four SNPs map near or in genes implicated in mechanisms modulating 25OHD levels, and, more specifically, transport (*GC*), synthesis (*DHCR7*), hepatic hydroxylation (*CYP2R1*), and catabolism (*CYP24A1*) [35].

**Table 1:** Characteristics of Single-Nucleotide Polymorphisms (SNPs) used as instrumental variables and their association with asthma, atopic dermatitis, and Immunoglobulin E (IgE) levels.

Vitamin D (25OHD) results								Asthma results			Childhood asthma results§			Atopic dermatitis results∞			IgE results§		
Locus	25OHD associated SNP	EA	EAF	Effect on 25OHD*	*p*^#^	F-Statistic♮	Variance in 25OHD explained by each SNP (%)	OR (95% CI)	*p*	*n*	OR (95% CI)	*p*	*n*	OR (95% CI)	*p*	*n*	Beta (95% CI)	*p*	*n*
CYP2R1	rs10741657	C	0.62	−0.052	3.3 x 10^−20^	18.78	0.13	0.99 (0.97–1.01)	0.54	142,551	1.02 (0.96–1.07)	0.56	15,008	1.02 (0.99–1.05)	0.27	40,834	−0.02 (−0.23 to 0.19)	0.86	12,853
DHCR7	rs12785878	G	0.27	−0.056	2.1 x 10^−27^	18.29	0.12	1.01 (0.98–1.03)	0.64	142,551	0.95 (0.90–1.01)	0.11	15,008	1.02 (0.98–1.06)	0.32	40,834	−0.15 (−0.36 to 0.06)	0.20	12,853
GC	rs2282679	C	0.3	−0.047	1.9 x 10^−109^	13.38	0.09	1.01 (0.99–1.04)	0.31	144,243	1.00 (0.95–1.06)	0.96	15,008	0.98 (0.94–1.02)	0.32	40,531	0.06 (−0.17 to 0.29)	0.60	12,853
CYP24A1	rs6013897	A	0.19	−0.027	6.0 x 10^−10^	3.13	0.02	1.02 (0.99–1.05)	0.14	144,243	1.03 (0.97–1.10)	0.38	15,008	1.03 (0.99–1.07)	0.22	40,529	0.02 (−0.25 to 0.29)	0.90	12,853

250HD, 25-hydroxyvitamin D; 95% CI, 95% confidence interval; EA, effect allele; EAF, effect allele frequency.

*Effect on natural log-transformed 25OHD levels in the Canadian Multicentre Osteoporosis Study (CaMos) Cohort, adjusted for age, age^2^, sex, season of blood draw, and body mass index (BMI).

^#^*p*-Values derived from the Study of Underlying Genetic Determinants of Vitamin D and Highly Related Traits (SUNLIGHT) Consortium.

^♮^F-Statistic derived from multiply adjusted natural log-transformed 25OHD levels in the CaMos Cohort.

^**♭**^Results are derived from the meta-analysis of the UK Biobank study and the GABRIEL asthma consortium.

^§^ Results are derived from the GABRIEL asthma consortium.

^∞^ Results are derived from the Early Genetics and Lifecourse Epidemiology (EAGLE) Eczema Consortium.

#### LD, confounding, and pleiotropy assessment

We found no evidence of LD between any of these SNPs (all pairwise *r*^*2*^ ≤ 0.01).

In our literature search for potential confounders, obesity and smoking were identified as risk factors for asthma [36] that have been associated with vitamin D levels [30]. We found no association between these SNPs and BMI (all *p*-values ≥ 0.29) in the Genetic Investigation of Anthropometric Traits (GIANT) [37] consortium or with smoking in the Tobacco and Genetics Consortium [38] (all *p*-values ≥ 0.18) (S1 Table).

**Fig 1:**
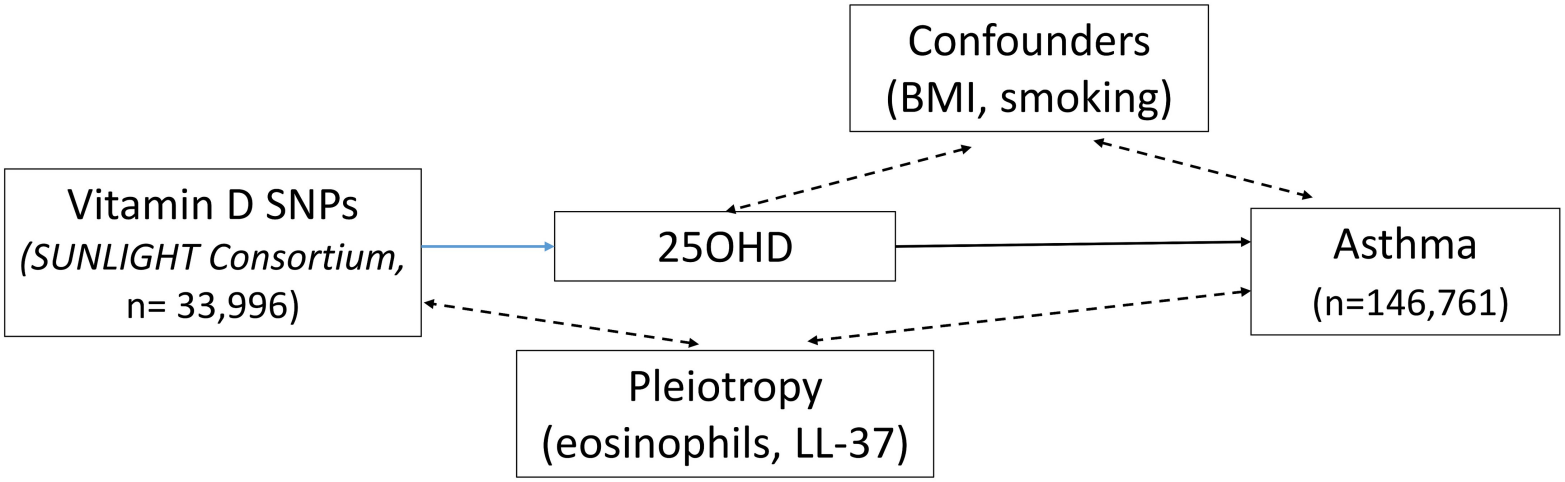
Direct Acyclic Graph (DAG) of the Mendelian randomization analysis for asthma. The effect of single-nucleotide polymorphisms (SNPs) on the change in natural log-transformed 25-hydroxyvitamin D (25OHD) levels. BMI, body mass index; SUNLIGHT, Study of Underlying Genetic Determinants of Vitamin D and Highly Related Traits.

**Fig 2:**
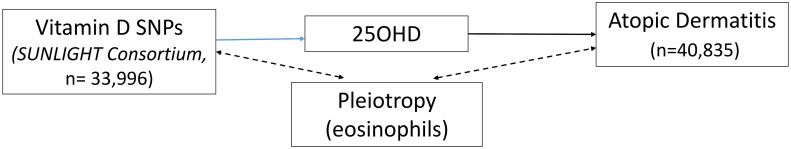
Direct Acyclic Graph (DAG) of the Mendelian randomization analysis for atopic dermatitis. The effect of single-nucleotide polymorphisms (SNPs) on the change in natural log-transformed 25-hydroxyvitamin D (25OHD) levels. SUNLIGHT, Study of Underlying Genetic Determinants of Vitamin D and Highly Related Traits.

**Fig 3:**
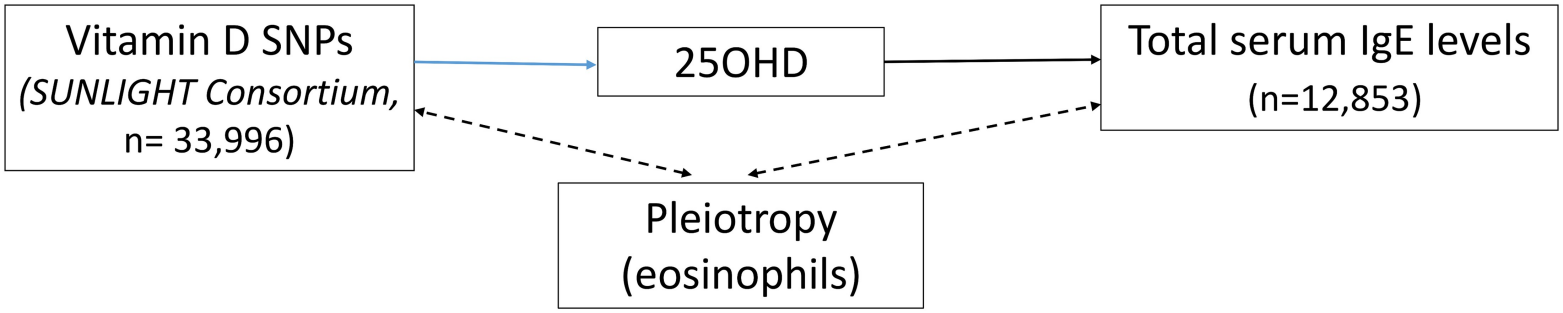
Direct Acyclic Graph (DAG) of the Mendelian randomization analysis for Immunoglobulin E (IgE) levels. The effect of single-nucleotide polymorphisms (SNPs) on the change in natural log-transformed 25-hydroxyvitamin D (25OHD) levels. SUNLIGHT, Study of Underlying Genetic Determinants of Vitamin D and Highly Related Traits.

The 25OHD-associated SNPs may also influence risk of atopic disease, independently of 25OHD, through pleiotropy (Figs 1, 2, and 3). Two *CYP2R1* SNPs (rs2060793 and rs1933064) have been associated with increased eosinophil counts, while the *GC* SNP rs7041 and the *CYP2R*1 SNP rs7935792 may be associated with changes in total IgE levels [39]. Therefore, to investigate possible pleiotropy, we tested the *CYP2R1* SNP rs10741657 for LD with the aforementioned *CYP2R1* SNPs. We found evidence for strong LD between the SUNLIGHT SNP rs10741657 and the eosinophil-related rs2060793 (*r*^*2*^ = 0.96), but no evidence for linkage between rs10741657 and the two other SNPs *(r*^*2*^ < 0.2). We also found weak LD between the SUNLIGHT *GC* SNP (rs2282679) and rs7041, which is associated with IgE levels (*r*^*2*^ = 0.5). Additionally, our literature review found evidence of an association in children between *CYP24A1* mRNA and LL-37, an immunomodulating peptide potentially related to asthma [29]. Therefore, we performed sensitivity analyses excluding the *CYP2R1* and *CYP24A1* SNPs (rs10741657 and rs6013897, respectively) from our MR instruments in our asthma analysis and excluding the *CYP2R1* SNP (rs10741657) in our atopic dermatitis and IgE analysis.

#### Population stratification assessment

Based on our previously published results [26], only rs12785878 at *DHCR7* was strongly associated with non-European ancestry. Given that the prevalence of both asthma and atopic dermatitis is increased in the individuals of African ancestry [40], we undertook sensitivity analyses excluding this SNP.

### Association of SUNLIGHT SNPs with 25OHD levels

The association of the four genome-wide significant SNPs from SUNLIGHT with 25OHD levels is described in Table 1. The proportion of the population-level variance in 25OHD levels explained by the four SNPs is reflected by the F-statistics. Although the low F-statistic of the *CYP24A1* SNP suggests that this might be a rather “weak” MR instrument, it is important to note that these F-statistics are derived from CaMos, a subsample of the SUNLIGHT study, and consequently the F-statistics would tend to increase if they were tested in the entire SUNLIGHT study. We have previously shown [41] that the count of 25OHD decreasing alleles across these four SNPs was strongly associated with lower total 25OHD levels in CaMos (*p* = 2.4 x 10^−12^) (Table 1).

### Association of SUNLIGHT SNPs with asthma and atopic dermatitis susceptibility and IgE levels

Summary statistics for the four 25OHD-associated SNPs and their associations with asthma were taken from the fixed-effects meta-analysis of the UK Biobank and GABRIEL studies. Since the *CYP24A1* SNP was absent in the UK Biobank genotypic dataset, we used the estimate of its perfect proxy rs17217119 (*r*^*2*^ = 1.0). None of the four 25OHD-decreasing alleles were associated with risk of asthma, childhood asthma, atopic dermatitis, or elevated IgE levels (Table 1), and the 95% confidence intervals were generally tight around the null.

### MR analysis for the association of 25OHD with asthma risk

**Fig 4:**
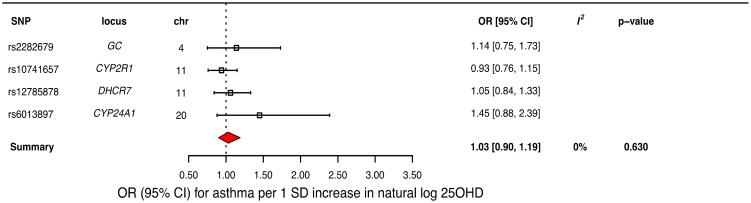
Mendelian randomization estimate of the association of 25-hydroxyvitamin D (25OHD) levels with risk of asthma. Estimates obtained from using a fixed-effects model. 95% CI, 95% confidence interval; chr, chromosome, OR, odds ratio; SD, standard deviation; SNP, single-nucleotide polymorphism.

In order to estimate the association of genetically lowered 25OHD with asthma, we used a fixed-effects model including all four 25OHD-decreasing alleles. A decrease in 25OHD levels by one SD on the natural log scale was not associated with asthma (odds ratio [OR] = 1.03, 95% CI 0.90–1.19; *p* = 0.63, *I*^*2*^ = 0%) (Table 2 and Fig 4). Since our model included only four SNPs, the 95% CIs of the *I*^*2*^ statistic were wide (0%–85%) and, consequently, heterogeneity could not be accurately measured using this parameter. In addition, because of potential population stratification, we undertook sensitivity analyses by excluding the *DHCR7* SNP and again observed no association with asthma (Table 3). To assess the effect of the independent vitamin D pathways on risk of asthma, we analyzed SNPs near genes implicated in 25OHD synthesis (*DHCR7* and *CYP2R1*) and metabolism (*GC* and *CYP24A1*) separately and found that none were associated with increased risk of asthma (Table 3). Also, because of evidence of possible pleiotropy for the *CYP2R1* and *CYP24A1* SNPs, we performed a sensitivity analysis after removing these two variants. The results again showed no evidence of an effect (Table 3).

**Table 2:** Mendelian Randomization (MR) estimates of the association of decreased 25-hydroxyvitamin D (25OHD) on the risk of asthma, atopic dermatitis, and elevated Immunoglobulin E (IgE) levels.

Outcome	MR estimate odds ratio or beta (95% CI)	*p*	I^2^ (95% CI)
Asthma	1.03 (0.90–1.19)*	0.63	0% (0%–85%)
Childhood asthma	0.95 (0.69–1.31)*	0.76	0% (0%–85%)
Atopic dermatitis	1.12 (0.92–1.37)*	0.27	15% (0%–87%)
IgE levels	−0.40 (−1.65 to 0.85)**	0.54	0% (0%–85%)

95% CI, 95% confidence interval.

*Odds ratio (OR) is expressed as the odds of asthma or atopic dermatitis per standard deviation decrease in natural log-transformed 25OHD levels.

**Beta is the effect per standard deviation decrease in natural log-transformed 25OHD levels on natural log-transformed total IgE levels.

**Table 3:** Sensitivity analyses testing Mendelian Randomization (MR) assumptions.

	Asthma		Atopic dermatitis		IgE levels	
Sensitivity analysis model	OR (95% CI) *	*p*	OR (95% CI) *	*p*	Beta (95% CI) **	*p*
Excluding the *DHCR7* locus	1.02 (0.86–1.22)	0.8	1.08 (0.85–1.39)	0.53	0.17 (−1.41 to 1.75)	0.83
Synthesis loci (*CYP2R1* and *DHCR7*)	0.99 (0.85–1.15)	0.89	1.20 (0.94–1.52)	0.14	−0.80 (−2.28 to 0.68)	0.29
Metabolism loci (*GC* and *CYP24A1*)	1.20 (0.96–1.51)	0.12	0.95 (0.65–1.37)	0.77	0.63 (−1.73 to 2.99)	0.60
Excluding the *CYP2R1* and *CYP24A1* loci	1.09 (0.92–1.30)	0.31	N/A	N/A	N/A	N/A
Excluding the *CYP2R1* locus	N/A	N/A	1.07 (0.83–1.38)	0.27	−0.50 (−2.04 to 1.04)	0.53

“N/A” is denoted when potential bias was not detected for the analysis. 95% CI, 95% confidence interval; IgE, immunoglobulin E.

*Odds ratio (OR) is expressed as the odds of asthma or atopic dermatitis per standard deviation decrease in natural log-transformed 25-hydroxyvitamin D (25OHD) levels.

**Beta is the effect per standard deviation decrease in natural log-transformed 25OHD levels on natural log-transformed total IgE levels.

Testing the effect of genetically reduced 25OHD on risk of childhood asthma, we found that each SD decrease in natural log-transformed 25OHD was not associated with risk of childhood asthma (OR = 0.95, 95% CI 0.69–1.31; *p* = 0.76, *I*^*2*^ = 0%).

Given these null results, we undertook a power calculation [34]. Based on a clinically relevant effect of an OR of 1.6 for asthma [1], a sample size of 144,243 individuals, and setting alpha to 0.05, our study had a power of 100% to detect an OR of 1.6 for a 1 SD change in log-transformed 25OHD levels on asthma risk, and an 80% power to exclude effects as small as an OR of 1.12. The same power calculation for a sample size of 15,008 individuals for childhood asthma gave to our study 100% power to detect an OR of 1.6 for a 1 SD change in log-transformed 25OHD levels on childhood asthma risk, and an 80% power to exclude effects as small as an OR of 1.33.

### MR analysis of the association of 25OHD with atopic dermatitis

**Fig 5:**
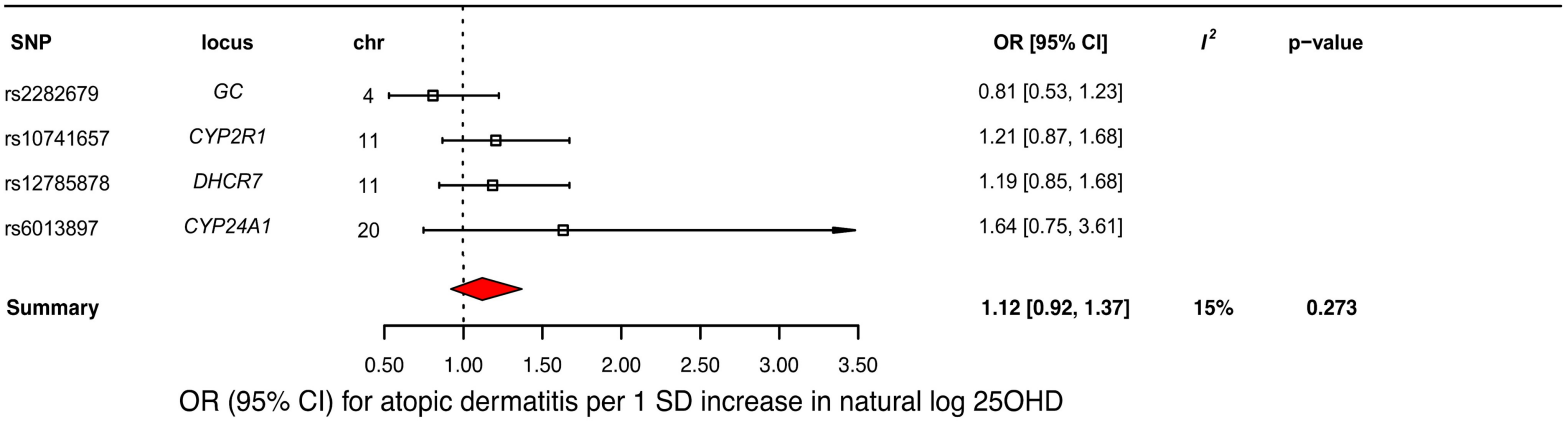
Mendelian randomization estimate of the association of 25-hydroxyvitamin D 25OHD levels with risk of atopic dermatitis. Estimates obtained from using a fixed-effects model. 95% CI, 95% confidence interval; chr, chromosome, OR, odds ratio; SD, standard deviation; SNP, single-nucleotide polymorphism.

A decrease in 25OHD levels by 1 SD on the natural log scale was not associated with atopic dermatitis (OR = 1.12, 95% CI 0.92–1.37; *p* = 0.27, *I*^*2*^ = 15%) (Table 2 and Fig 5). Similar to the previous analysis for asthma, we undertook sensitivity analyses by excluding the *DHCR7* SNP to control for possible population stratification and by removing the *CYP2R1* SNP, because of potential pleiotropy, and the results were similar (Table 3). Analyzing SNPs near genes implicated in 25OHD synthesis and metabolism separately, again no association was found (Table 3). Based on a previously reported OR of 1.5 for atopic dermatitis in vitamin D insufficient individuals [5], a sample size of 40,835 individuals, and setting alpha to 0.05, our study had a power of 100% to detect an OR of 1.5 for 1 SD decrease in log-transformed 25OHD levels on atopic dermatitis risk, and an 80% power to observe effects down to an OR of 1.21.

### MR analysis of the association of 25OHD with IgE levels

**Fig 6:**
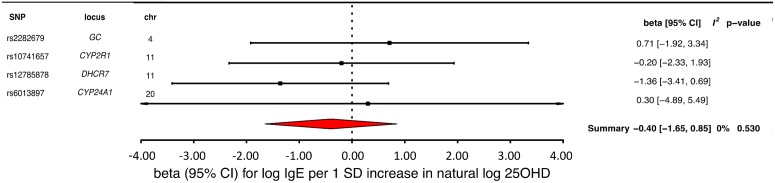
Mendelian randomization estimate of the association of 25-hydroxyvitamin D (25OHD) levels with Immunoglobulin E (IgE) levels. Estimates obtained from using a fixed-effects model. 95% CI, 95% confidence interval; chr, chromosome, OR, odds ratio; SD, standard deviation; SNP, single-nucleotide polymorphism.

MR analyses for IgE levels showed that a decrease in 25OHD levels by 1 SD on the natural log scale was not associated with an increase in naturally log-transformed total serum IgE levels (beta = −0.40 natural log-transformed units, 95% CI −1.65 to 0.85, *p* = 0.54, *I*^*2*^ = 0) (Table 2 and Fig 6). After sensitivity analyses excluding the *DHCR7* or the *CYP2R1* SNP, and analyzing separately 25OHD synthesis and metabolism SNPs, the results still included the null (Table 3). Based on a previously reported beta of −0.43 for log-transformed total IgE levels per SD increase in log-transformed 25OHD levels [42], our sample size of 12,853 individuals, and setting alpha to 0.05, our study had a power of 86%.

## Discussion

Using large populations of individuals of European descent, our study failed to provide evidence supporting a role for vitamin D in adult and childhood onset asthma, atopic dermatitis, and IgE levels, although small effects cannot be excluded. These findings provide no rationale for the use of vitamin D supplementation for prevention of these conditions in populations of similar ethnicity.

Residual confounding may account for a large part of the discrepancy between our findings and those of observational studies. For instance, adiposity predisposes to asthma [43] and lowers 25OHD levels [44]. While previous studies have controlled for BMI, it is a poor measure of adiposity [45]. Physical activity might also be a strong confounder in observational studies, because it is associated with asthma [46] and sunlight exposure, which in turn influences vitamin D status. Another possible explanation of the discrepancy between MR results and observational studies could be reverse causation, since asthmatic individuals tend to be less active [46] and are therefore less exposed to sunlight. Further, steroid therapies used in asthma may result in low vitamin D levels [47]. Last, individuals with darker skin are at increased risk of atopic dermatitis and asthma [40], while they are also more susceptible to develop vitamin D insufficiency [48].

Our results are in accordance with two recent meta-analyses of RCTs [15,16], which conclude that evidence is lacking to support regular use of vitamin D supplements for prevention of asthma exacerbations, but are in contrast with the findings of a recent Cochrane meta-analysis of RCTs [14]. Our findings for atopic dermatitis and IgE agree with recent observational studies and RCTs [3,4,9]. In contrast, we have used the same methods to provide evidence supporting a causal role for 25OHD in risk of multiple sclerosis [41]. Thus, it would appear that immunomodulatory effects of 25OHD do not uniformly influence immune-mediated diseases.

Previous studies have explored the effects of vitamin D-related genes and risk on atopic phenotypes, without applying an MR approach [7,39,49–52]. SNPs in the vitamin D pathway (*CYP27A1*, *CYP27B1*, *CYP2R1*, *CYP24A1*, and *GC*) affecting 25OHD levels demonstrated moderate effects on risk of asthma in a prior adult study [49], but the role of these variants on asthma risk later in life is unknown. A recent study provided evidence of an association between the vitamin D receptor genes and asthma in adolescents with normal 25OHD levels [50]. Another study reported an association between a *CYP2R1* variant and FEV1 in children, and between specific haplotypes on *CYP2R1* and *CYP27A1* and asthma phenotypes [51]. With regard to atopic dermatitis, a polymorphism in the *CYP24A1* gene has been associated with severe atopic dermatitis in adults [52]. A vitamin D-related SNP on *CYP27A1* was also found to be protective against eczema, whereas *CYP2R1* and *VDR* haplotypes appear to alter eczema susceptibility. In regard to IgE levels, carrying a rare variant on *CYP27A1* appears to increase the risk of elevated total serum IgE levels (above 1000 IU/ml), and a *CYP27B1* allele has also been associated to IgE levels [53]. Nevertheless, other than testing genetic associations, the above studies were not designed to test the causal relationship between 25OHD levels and atopy.

Other MR studies have been carried out in asthma and have provided evidence supporting a causal role for increased BMI in asthma [43], and evidence against a causal role of prenatal alcohol exposure in asthma and atopy in childhood [54]. A recent MR study used a smaller sample of 1,208 cases and 3,877 controls for childhood asthma in individuals of European and non-European ancestry [55] and did not find any evidence for a causal role of vitamin D in asthma. The instruments used in this childhood MR asthma study were only the *CYP2R1* and *GC* SNPs and did not include the SNPs near *DHCR7* and *CYPR24A1*. Our study thus provides a more thorough examination of the effects of 25OHD on asthma risk by using a substantially larger sample size, including all 25OHD-associated loci, in both adult and childhood asthma.

Strengths of this study include the large sample size of the adult cohorts, which enabled us to more precisely test our study hypothesis than if we had used individual-level data from small studies. Although the findings from this study were null, the high statistical power and tight confidence intervals exclude most clinically relevant effects of 25OHD on risk of asthma, atopic dermatitis, and IgE levels. Importantly, our findings represent the association of a life-long exposure to reduced vitamin D levels in the general population.

This study also has limitations. While we controlled for pleiotropy, residual bias is possible since the exact function of the SNPs studied is unknown. However, all the SNPs lie in, or near, genes well validated for their role in vitamin D physiology. The null result could also be explained by canalization, which is a phenomenon resulting in compensatory feedback mechanisms [20]. Our childhood asthma MR study was underpowered to exclude effects of vitamin D on pediatric asthma smaller than an OR less than 1.33 per SD change in log vitamin D. There was heterogeneity in the definition of the different atopic outcomes, since in some GWAS used for this MR study the outcomes were self-reported, and in others physician-diagnosed (see S1 Text). Our MR analysis might also be limited in its ability to elucidate a possible role of biologically active vitamin D, reflected by the levels of the active metabolite 1,25-dihydroxyvitamin D. Although genetically lowered total 25OHD levels do not appear to be associated with increased risk of the studied atopic phenotypes, we have not assessed whether reduced lifelong 1,25-dihydroxyvitamin D could influence these outcomes, since levels of total 25OHD and 1,25-dihydroxyvitamin D are weakly correlated [56]. Also, our study can only address the role of circulating 25OHD levels, and not on the action of 25OHD at the cellular level. Our analyses were restricted to white populations of European ancestry, and further work will be required to investigate their relevance in populations of different ethnicity or in those with frank vitamin D deficiency.

In conclusion, our MR study provides no support for an unconfounded relationship between 25OHD levels and risk of atopic disease in individuals of European descent. Instead, association of 25OHD levels with atopic diseases in the general population is more likely to be attributable to confounding by lifestyle factors such as obesity and physical inactivity.

## Supporting information

S1 Checklist Strengthening the Reporting of Observational Studies in Epidemiology (STROBE) checklist. (DOCX)

S1 Table *p*-Values of the association of the Single-Nucleotide Polymorphisms (SNPs) used as instrumental variables with potential confounders. (DOCX)

S1 Text Phenotype definition in the participating studies and PubMed search for pleiotropy. (DOCX)

## Abbreviations

25OHD: 25-hydroxyvitamin D
95% CI: 95% confidence interval
BMI: body mass index
CaMos: Canadian Multicentre Osteoporosis Study
DAG: direct acyclic graph
EAGLE: Early Genetics and Lifecourse Epidemiology
GIANT: Genetic Investigation of Anthropometric Traits
GWAS: genome-wide association study
IgE: immunoglobulin E
LD: linkage disequilibrium
MR: Mendelian randomization
OR: odds ratio
RCT: randomized controlled trial
SD: standard deviation
SNP: single-nucleotide polymorphism
STROBE: Strengthening the Reporting of Observational Studies in Epidemiology
SUNLIGHT: Study of Underlying Genetic Determinants of Vitamin D and Highly Related Traits
