# Supplementary material for: Vitamin D levels and susceptibility to asthma, elevated immunoglobulin E levels, and atopic dermatitis: A Mendelian randomization study
Source: PLoS Med. 2017 May 9;14(5):e1002294. doi: 10.1371/journal.pmed.1002294 (PMC5423551; doi:10.1371/journal.pmed.1002294)
Supplement: S1 Table — (DOCX) [file pmed.1002294.s002.docx]

**S1 Table: P-values of the association of the SNPs used as instrumental variables with potential confounders.**

| **25OHD-SNP Look-up in GWAS Consortia** | | | | |
| --- | --- | --- | --- | --- |
| **Trait** | **p for association with *GC***  **(rs2282679)** | **p for association with *CYP2R1***  **(rs10741657)** | **p for association**  **with *DHCR7* (rs12785878)** | **p for association with *CYP24A1* (rs6013897)** |
| BMI^a^ | 0.91 | 0.29 | 0.78 | 0.61 |
| Smoking Quantity^b^ | 0.80 | 0.90 | 0.18 | 0.73 |

^a^  p-value for association between 25OHD SNP and BMI obtained from the GIANT Consortium [1].

^b^p-value for association between 25OHD SNP and smoking quantity obtained from the Tobacco and Genetics Consortium [2].

References

1. Manning AK, Hivert MF, Scott RA, Grimsby JL, Bouatia-Naji N, Chen H, et al. A genome-wide approach accounting for body mass index identifies genetic variants influencing fasting glycemic traits and insulin resistance. Nat Genet. 2012;44(6):659-69. doi: 10.1038/ng.2274. PubMed PMID: 22581228; PubMed Central PMCID: PMCPMC3613127.

2. Tobacco, Genetics C. Genome-wide meta-analyses identify multiple loci associated with smoking behavior. Nat Genet. 2010;42(5):441-7. doi: 10.1038/ng.571. PubMed PMID: 20418890; PubMed Central PMCID: PMCPMC2914600.
