# Supplementary material for: Vitamin D levels and susceptibility to asthma, elevated immunoglobulin E levels, and atopic dermatitis: A Mendelian randomization study
Source: PLoS Med. 2017 May 9;14(5):e1002294. doi: 10.1371/journal.pmed.1002294 (PMC5423551; doi:10.1371/journal.pmed.1002294)
Supplement: S1 Text — (DOCX) [file pmed.1002294.s003.docx]

**S1 Text**

*Phenotype Definition in the Participating Studies*

The UK Biobank Asthma GWAS was conducted using baseline phenotypic data collected in the UK Biobank study. UK Biobank^1^ comprises ~500,000 people aged between 40-69 years, recruited between 2006 and 2010 across Great Britain.^2^ Genotypic data were available in 120,286 UK Biobank participants with known asthma status (15,106 cases/105,180 controls). Asthma was defined as self-reported asthma, without further specification on the age of asthma onset.

The GABRIEL study is a meta-analysis of 37 GWAS studies of individuals of European descent (10,003 cases/13,954 controls), of which 20 studies included individuals with childhood-onset asthma (7,047 cases/7,961 controls), aiming to identify risk factors for asthma.^3^ Asthma was considered present if diagnosed by a physician. Childhood-onset asthma was defined as onset of asthma before the age of 16 years. After quality control and filtering, genotypes were retained on 10,365 asthma cases and 16,110 controls from 37 different studies, including studies with both childhood onset and adult onset asthma. Only one of the four SUNLIGHT SNPs used as instruments for the MR was present in the published genotyped dataset of the GABRIEL consortium. Thus, we opted to use estimates on the effect of the four SUNLIGHT SNPs on asthma from a meta-analysis of Hapmap2-imputed GABRIEL data (unpublished), comprising all 37 studies of the original GABRIEL meta-analysis. We used a meta-analysis of data imputed in the same panel for the 20 childhood-onset asthma studies for our secondary analysis. The UK Biobank and GABRIEL summary level results for the four SNPs were then pooled in a fixed-effects meta-analysis using the GWAMA software^4^.

For atopic dermatitis, we obtained the effects of the four SUNLIGHT 25OHD-associated SNPs on this disease using data from The EAGLE Eczema Consortium^5^. EAGLE is a large meta-analysis of 26 studies, including 116,863 individuals (21,399 cases/95,464 controls), imputed to the 1000 Genomes Project Phase 1 reference panel. The data used in this MR study were obtained from the fixed-effect meta-analysis of 22 European studies (excluding 23andMe). This resulted in a sample of 10,788 cases and 30,047 controls. Phenotype definition of atopic dermatitis differed among studies and was either self-reported or based on dermatological exam.

For IgE levels, we obtained the effects for the four SUNLIGHT SNPs from 34 of the 37 studies of the GABRIEL consortium. The GWAS for IgE was conducted separately in studies with asthma cases only (total n=5,888) and in studies with controls only (n=6,965), and the results were combined by fixed-effects meta-analysis within cases (17 datasets) and within controls (17 datasets). The combination of these two pooled results, inversely weighted for their variance, is arithmetically equivalent to doing a single fixed-effects meta-analysis of all the study-stratum-specific results (from 34 datasets) in one calculation. Therefore the betas from this analysis represent the pooled estimates of the effect of the SNPs on naturally log transformed total IgE, derived from a fixed-effects (inverse-variance-weighted) meta-analysis of the results from the asthmatics and from the controls.

*PubMed Search for Pleiotropy*

The following terms were searched on the PubMed database to investigate possible pleiotropic mechanisms of our chosen SNPs corresponding to gene name, gene mutations, encoded protein, encoded protein with asthma, atopic dermatitis and IgE levels.

For rs2282679: “GC”, “GC gene”, “GC gene mutations”, “vitamin D binding protein”, “vitamin D binding protein asthma”, “vitamin D binding protein atopic dermatitis”, “vitamin D binding protein IgE levels”

The search term GC uncovered 69152 results, most of which were not relevant to genetics, therefore the search term “GC gene” was used instead to refine search results.

For rs12785878: “DHCR7”, “DHCR7 mutations”, “7-dehydrocholesterol reductase”, “7-dehydrocholesterol reductase asthma”, 7-dehydrocholesterol reductase atopic dermatitis”, “7-dehydrocholesterol reductase IgE levels”.

For rs6013897: “CYP24A1”, “CYP24A1 mutations”, “1,25-dihydroxyvitamin D3 24-hydroxylase”, “1,25-dihydroxyvitamin D3 24-hydroxylase asthma” “1,25-dihydroxyvitamin D3 24-hydroxylase atopic dermatitis” ,“1,25-dihydroxyvitamin D3 24-hydroxylase IgE levels”

For rs10741657: “CYP2R1”, “CYP2R1 mutations”, “vitamin-D hydroxylase”, “vitamin-D hydroxylase asthma”, “vitamin-D hydroxylase atopic dermatitis”, “vitamin-D hydroxylase IgE levels”.

Abstracts were selected for further review if they made reference to the search term and a pathway distinct from vitamin D or vitamin D insufficiency/ deficiency on the three outcomes. Only studies in mammals were considered. Findings are reported in the Results section.

**REFERENCES**

1. Ollier W, Sprosen T, Peakman T. UK Biobank: from concept to reality. Pharmacogenomics 2005;6:639-46.

2. Sudlow C, Gallacher J, Allen N, et al. UK biobank: an open access resource for identifying the causes of a wide range of complex diseases of middle and old age. PLoS Med 2015;12:e1001779.

3. Moffatt MF, Gut IG, Demenais F, et al. A large-scale, consortium-based genomewide association study of asthma. N Engl J Med 2010;363:1211-21.

4. Magi R, Morris AP. GWAMA: software for genome-wide association meta-analysis. BMC Bioinformatics 2010;11:288.

5. Paternoster L, Standl M, Chen CM, et al. Meta-analysis of genome-wide association studies identifies three new risk loci for atopic dermatitis. Nat Genet 2012;44:187-92.
